# Supplementary material for: Pleistocene drivers of Northwest African hydroclimate and vegetation
Source: Nat Commun. 2022 Jun 21;13:3552. doi: 10.1038/s41467-022-31120-x (PMC9213457; doi:10.1038/s41467-022-31120-x)
Supplement: Supplementary file 1 — Supplementary Information [file 41467_2022_31120_MOESM1_ESM.pdf]

Supplementary Information:
Pleistocene drivers of Northwest African hydroclimate and vegetation

Nicholas A. O'Mara[1,2*], Charlotte Skonieczny[3], David McGee[4], Gisela Winckler[1,2], Aloys J.-M. Bory[5], Louisa I. Bradtmiller[6], Bruno Malaizé[7], Pratigya J. Polissar[8]

[1]Lamont-Doherty Earth Observatory, Columbia University, Palisades, NY, USA
[2]Department of Earth and Environmental Sciences, Columbia University, New York, NY, USA
[3]Université Paris-Saclay, CNRS, GEOPS, 91405, Orsay, France
[4]Department of Earth, Atmospheric and Planetary Sciences, Massachusetts Institute of Technology, Cambridge, MA, USA
[5]Université de Lille, CNRS, Université Littoral Cote d'Opale, UMR 8187, LOG, Laboratoire d'Océanologie et de Géosciences, Lille, France
[6]Department of Environmental Studies, Macalester College, St. Paul, MN, USA
[7]UMR CNRS 5805 EPOC, Université Bordeaux I, 33405 Talence, France
[8]Ocean Sciences Department, University of California, Santa Cruz, CA, USA

*Corresponding author: omara@ldeo.columbia.edu

Supplementary Figures

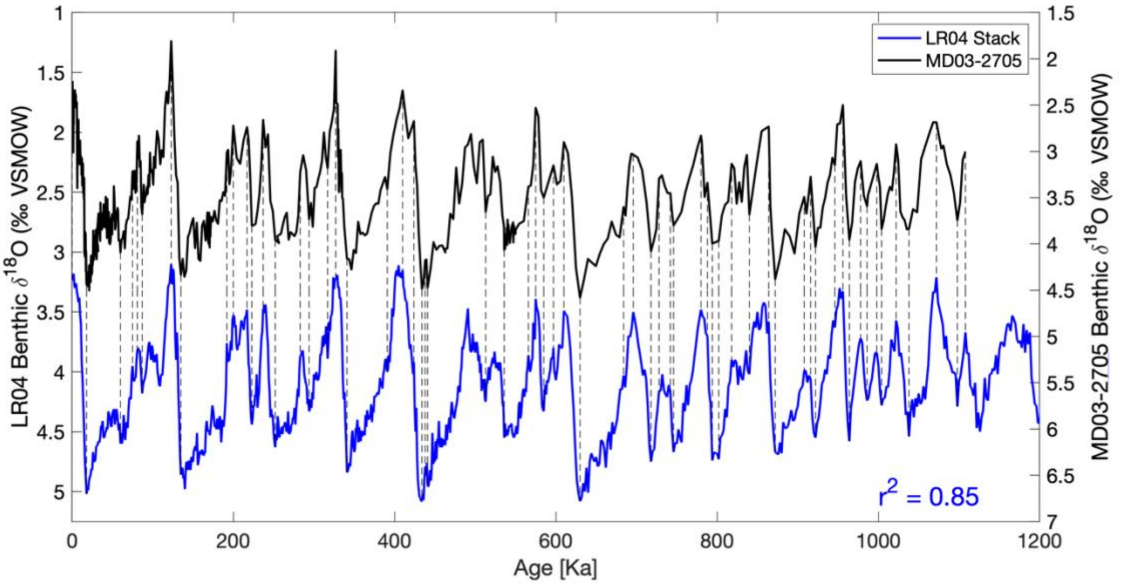

Figure S1. Benthic oxygen isotope stratigraphic tie points used to constrain the age model for core MD03-2705. The LR04 benthic $\delta^{18}O$ stack is plotted in blue[79], the benthic $\delta^{18}O$ record of MD03-2705 is plotted in black[78], with tie-lines indicated by dashed lines. Tie points can be found in Supplement Table 1.

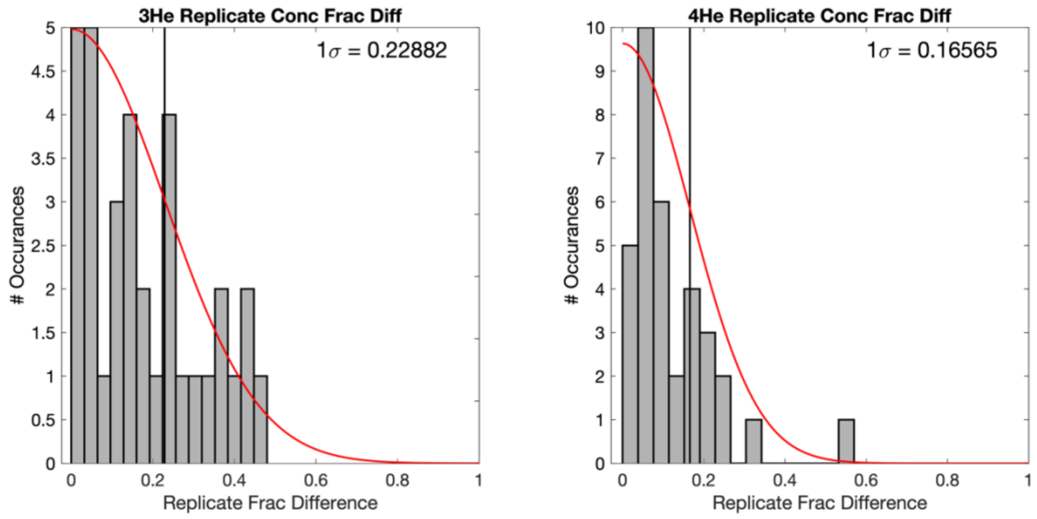

Figure S2. Distributions (histograms, gray; one-half Gaussian fits, red) of fractional differences between replicate measurements of sediment sample ^{3}He (left panel) and ^{4}He (right panel) concentrations. Standard deviations of the distributions (1σ) are plotted as black lines. These 1σ uncertainties are propagated to $^3He_{ET}$-normalized dust flux in the main text.

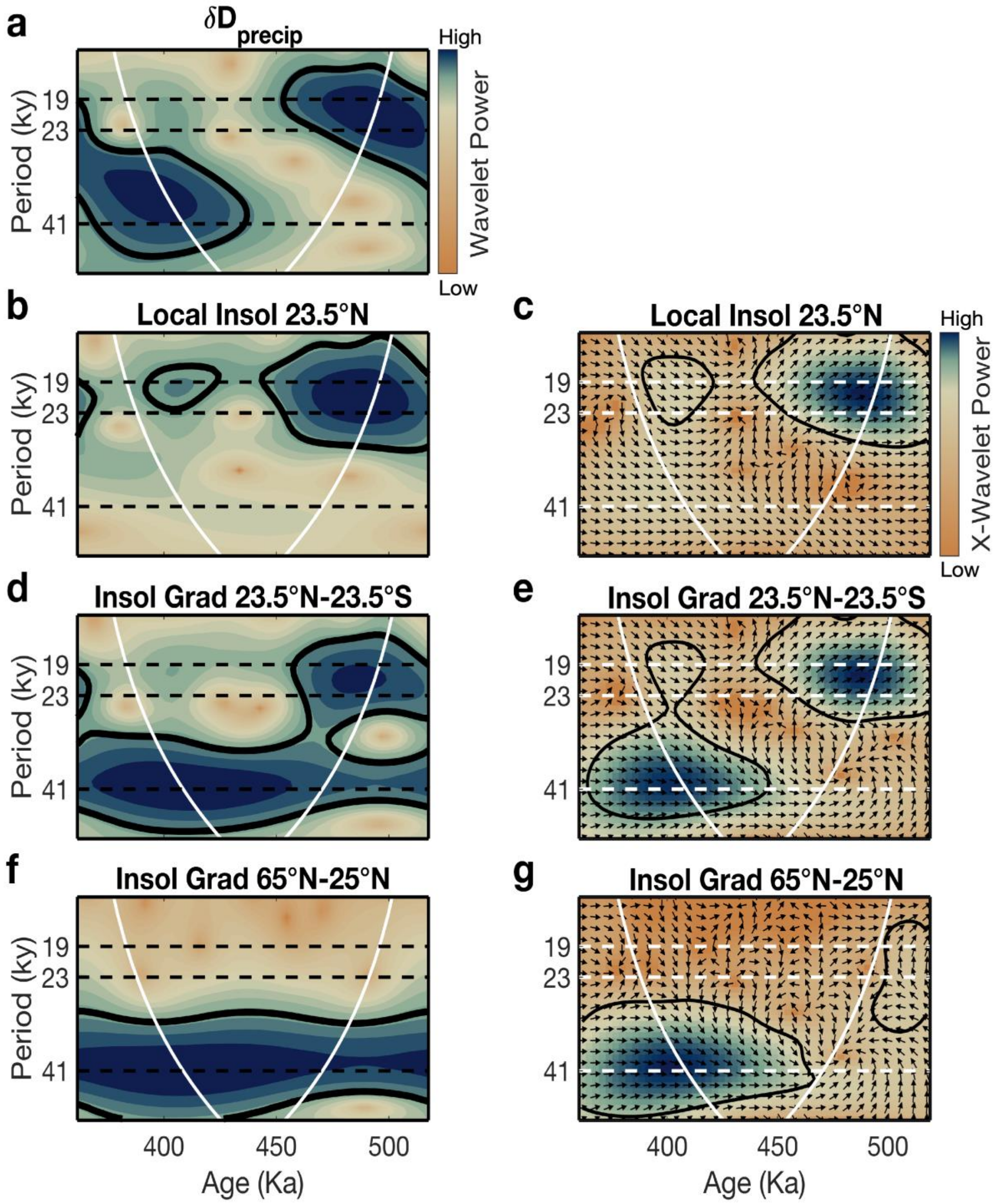

Figure S3. Wavelet and Cross wavelet analysis for MIS13–10 δD. Wavelet scalograms depicting power at different periods (colorbar) through time for **a.** δD_{precip} record, **b.** local summer insolation, 23.5°N June 21, **d.** summer inter-hemispheric insolation gradient 23.5°N – 23.5°S June 21, **f.** low to high-latitude summer insolation gradient, 25°N – 65°N June 21 (as in fig. 2). Cross-wavelet scalograms depicting cross-wavelet power (colorbar) and wavelet coherence shown in arrows (right, in phase; left, 180° out of phase; up, proxy lags forcing; down, proxy leads forcing) calculated using ref.[129] for δD_{precip} and **c.** local summer insolation, 23.5°N June 21, **e.** cross-equatorial summer insolation gradient, 23.5°N – 23.5°S June 21, and **g.** low to high-latitude summer insolation gradient, 25°N – 65°N June 21.

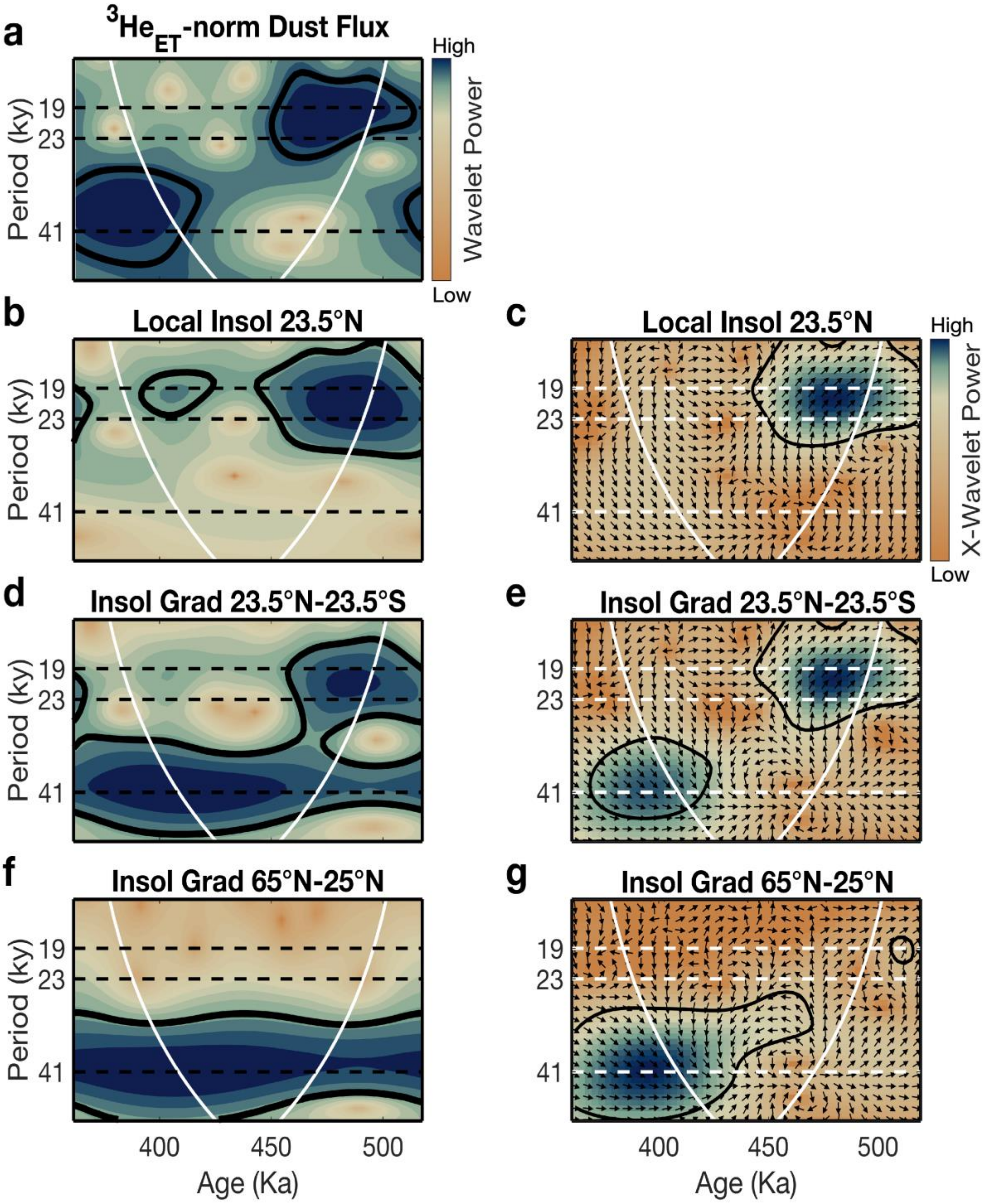

Figure S4. Wavelet and Cross wavelet analysis for MIS13–10 $^{3}He_{ET}$-normalized dust flux. Wavelet scalograms depicting power at different periods (colorbar) through time for **a.** $^{3}He_{ET}$-normlaized dust flux, **b.** local summer insolation, 23.5°N June 21, **d.** summer inter-hemispheric insolation gradient 23.5°N – 23.5°S June 21, **f.** low to high-latitude summer insolation gradient, 25°N – 65°N June 21 (as in fig. 2). Cross-wavelet scalograms depicting cross-wavelet power (colorbar) and wavelet coherence shown in arrows (right, in phase; left, 180° out of phase; up, proxy lags forcing; down, proxy leads forcing) calculated using ref.[129] for $^{3}He_{ET}$-normlaized dust flux and **c.** local summer insolation, 23.5°N June 21, **e.** cross-equatorial summer insolation gradient, 23.5°N – 23.5°S June 21, and **g.** low to high-latitude summer insolation gradient, 25°N – 65°N June 21.

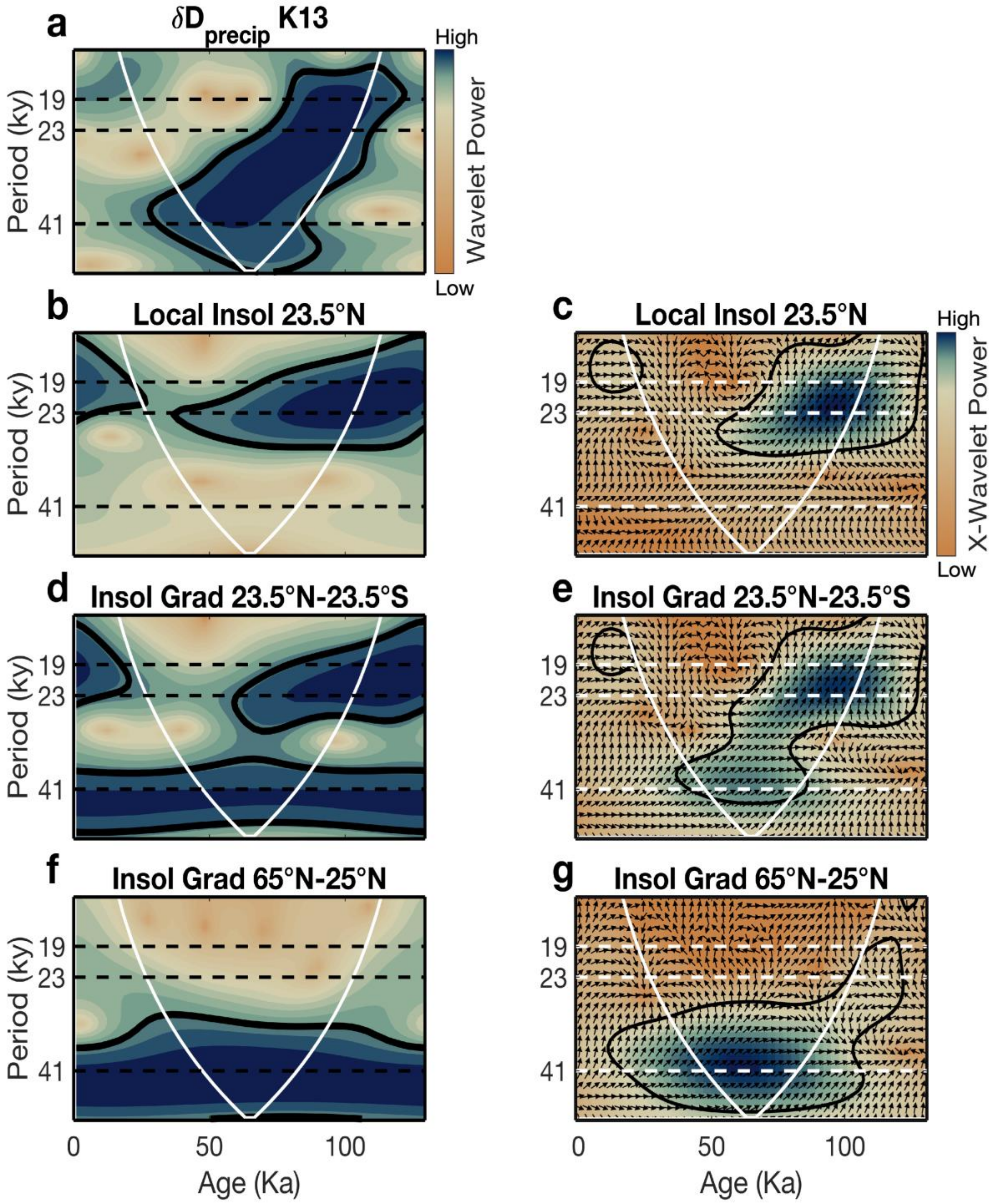

Figure S5. Wavelet and Cross wavelet analysis for MIS5-present δD. Wavelet scalograms depicting power at different periods (colorbar) through time for **a.** δD_{precip} record (ref.[42]), **b.** local summer insolation, 23.5°N June 21, **d.** summer inter-hemispheric insolation gradient 23.5°N – 23.5°S June 21, **f.** low to high-latitude summer insolation gradient, 25°N – 65°N June 21 (as in fig. 2). Cross-wavelet scalograms depicting cross-wavelet power (colorbar) and wavelet coherence shown in arrows (right, in phase; left, 180° out of phase; up, proxy lags forcing; down, proxy leads forcing) calculated using ref.[129] for δD_{precip} and **c.** local summer insolation, 23.5°N June 21, **e.** cross-equatorial summer insolation gradient, 23.5°N – 23.5°S June 21, and **g.** low to high-latitude summer insolation gradient, 25°N – 65°N June 21.

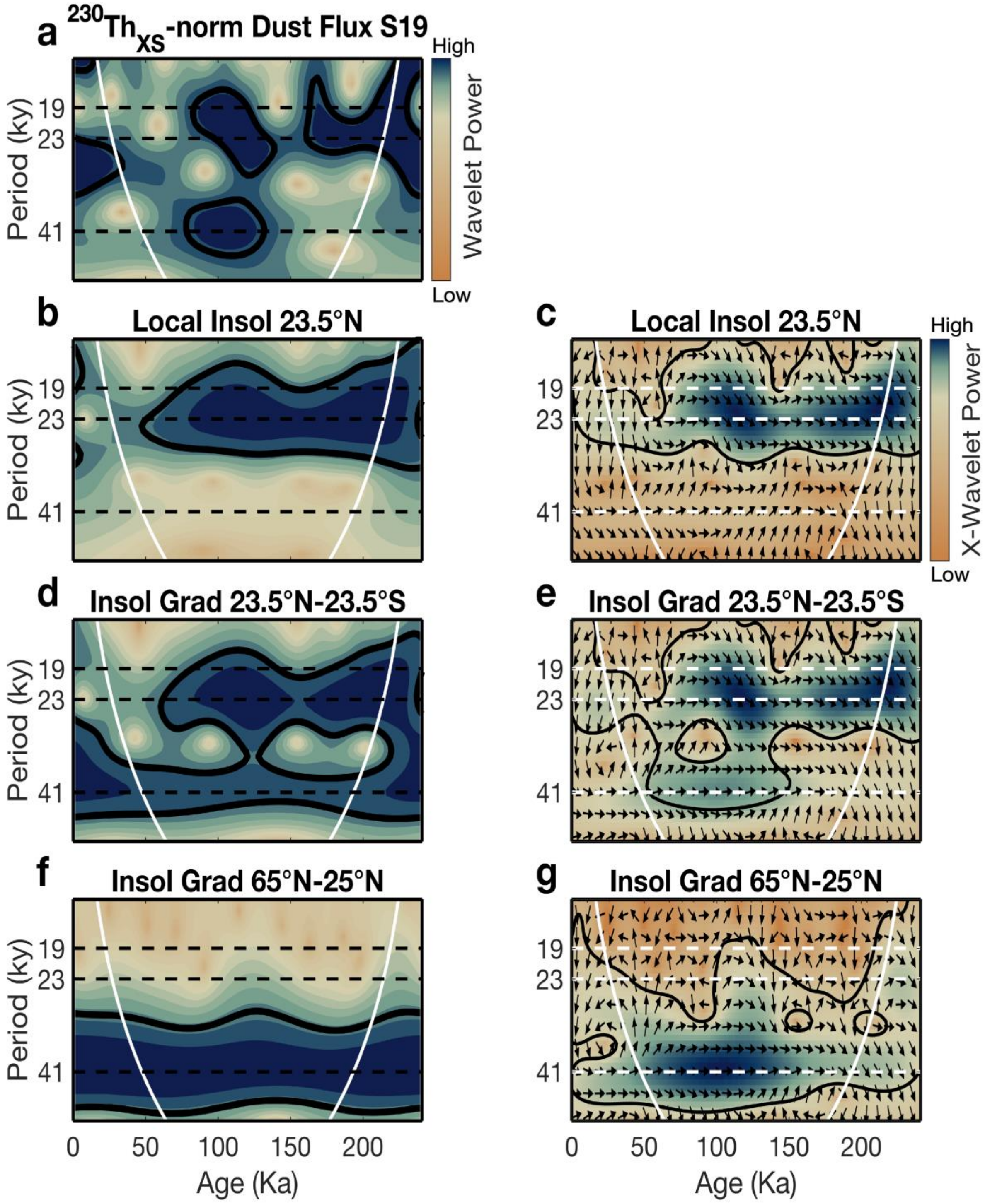

Figure S6. Wavelet and Cross wavelet analysis for MIS7–present $^{230}Th_{XS}$ -normalized dust flux. Wavelet scalograms depicting power at different periods (colorbar) through time for **a.** $^{230}Th_{XS}$-normlaized dust flux (ref.[20]), **b.** local summer insolation, 23.5°N June 21, **d.** summer inter-hemispheric insolation gradient 23.5°N – 23.5°S June 21, **f.** low to high-latitude summer insolation gradient, 25°N – 65°N June 21 (as in fig. 2). Cross-wavelet scalograms depicting cross-wavelet power (colorbar) and wavelet coherence shown in arrows (right, in phase; left, 180° out of phase; up, proxy lags forcing; down, proxy leads forcing) calculated using ref.[129] for $^{230}Th_{XS}$-normlaized dust flux and **c.** local summer insolation, 23.5°N June 21, **e.** cross-equatorial summer insolation gradient, 23.5°N – 23.5°S June 21, and **g.** low to high-latitude summer insolation gradient, 25°N – 65°N June 21.

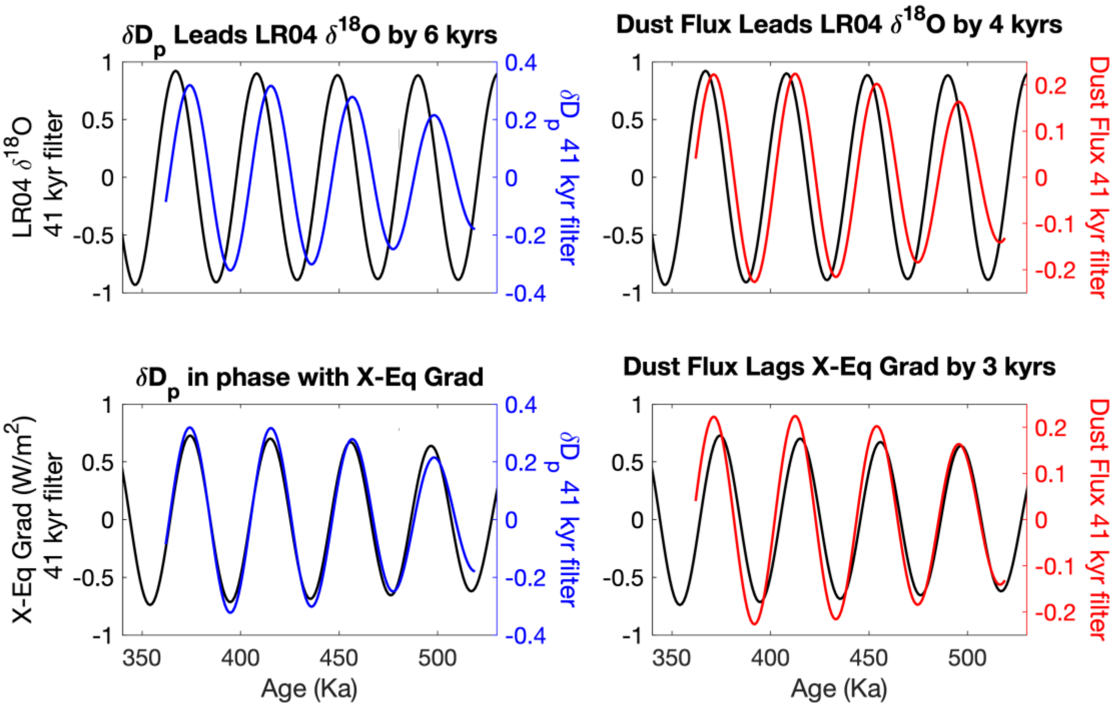

Figure S7. Obliquity forcing and response phase relationships. Comparison of 41 kyr bandpass filtered plant-wax δD_{precip} (first column, blue lines) and dust flux (second column, red lines) with two possible forcings of obliquity variability. The top row shows the LR04 $\delta^{18}O$ benthic stack while the bottom row cross equatorial insolation gradient (June 21 insolation at 23.5°N-23.5°S). Both forcings and proxies were filtered using the matlab script 'bandpass' to isolate obliquity variability at a 41 kyr period. Cross-correlations at 1 kyr lags were calculated to determine the phase relationship between the forcing and proxy response. Both δD_{precip} and dust flux are either in phase or lag the insolation forcing, while both proxies lead the ice volume forcing, precluding ice volume as the driver of the proxy responses at the period of orbital obliquity.

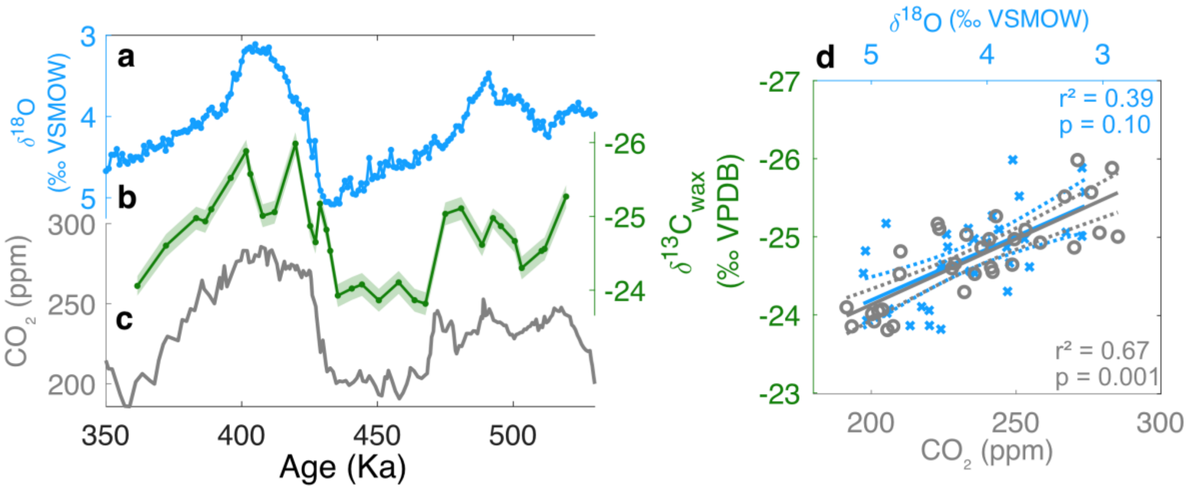

Figure S8. CO_2 vs. ice volume forcing of vegetation. a. LR04 benthic stack $\delta^{18}O$ (ref.[79]), **b.** $\delta^{13}C_{wax}$ (this study), **c.** atmospheric CO_2 concentrations (ref.[69]), **d.** linear correlations between (a) and (b) and (c) and (b) showing that CO_2 concentration in the atmosphere exerts a much stronger control on $\delta^{13}C_{wax}$ compared to changes in ice volume.

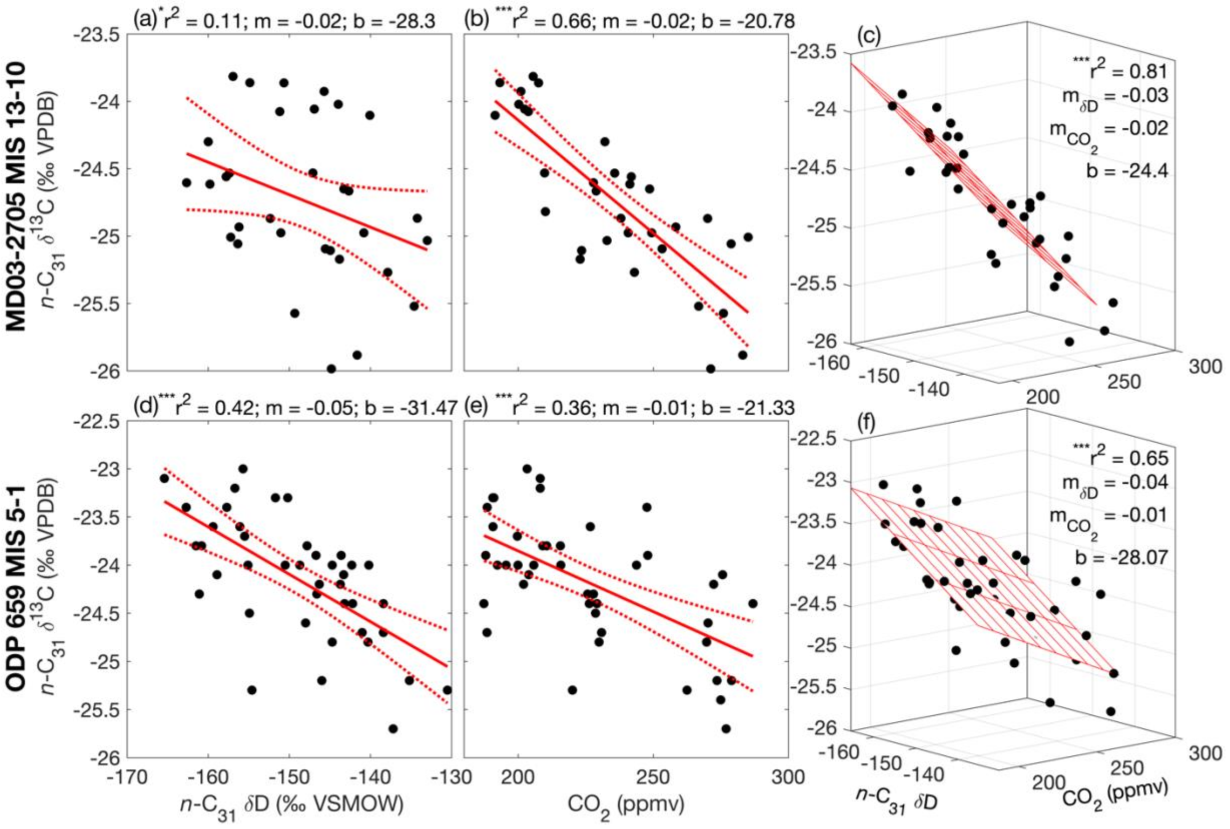

Figure S9. Plant wax and CO_2 regression analysis. Linear regressions of n-C_{31} $\delta^{13}C$ against n-C_{31} δD during **a.** MIS13-10 (this study), **d.** MIS5-present (ref.[42]) and atmospheric CO_2 (ref.[69]) during **b.** MIS13-10 (this study), **e.** MIS5-present (ref.[42]). Multiple linear regressions were calculated using the matlab script ‘regress’ with both n-C_{31} δD and CO_2 concentration as predictor variables of the n-C_{31} $\delta^{13}C$ values for **c.** MIS13-10 (this study), **f.** MIS5-present (ref.[42]). In all panels, regression statistics correlation coefficient (r^2), slope (m) and intercept (b) are shown.

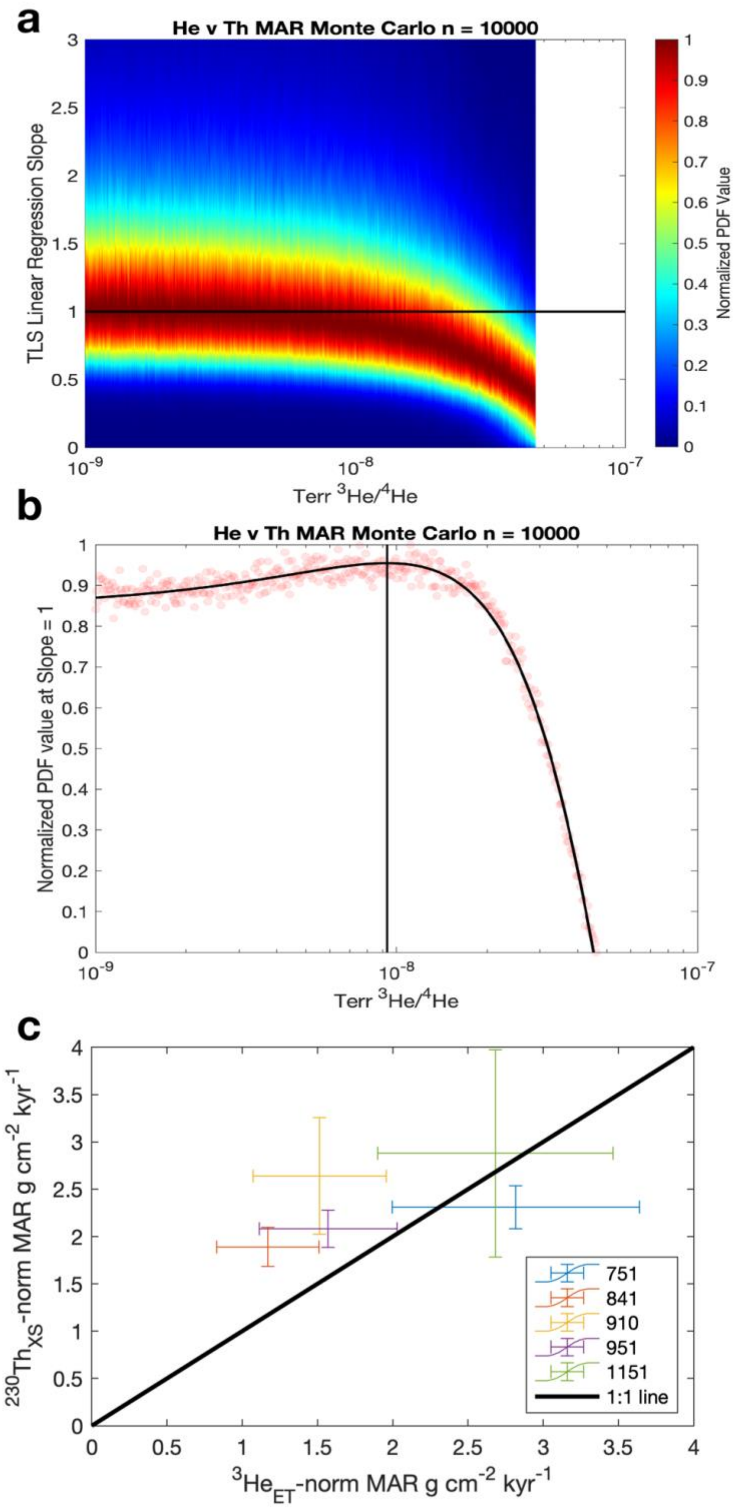

Figure S10. Monte Carlo end member modeling result. **a.** Terrestrial $^3He/^4He$ end-member values plotted against total least square linear regression slopes, colored by normalized PDF values of total least square linear regression slopes for each tested terrestrial $^3He/^4He$ end-member value. **b.** extracted values of the PDF from (a) at slope values equal to m = 1. The probability of a regression slope of m = 1 between the $^{230}Th_{XS}$- and $^3He_{ET}$-normalized dust fluxes is high for values of terrestrial $^3He/^4He$ end-member below values of $2x10^{-8}$, with a maximum close to $1x10^{-8}$ and with a sharp decline in probability values higher than $2x10^{-8}$. **c.** The total least squares linear regression between the $^{230}Th_{XS}$- and $^3He_{ET}$-normalized dust fluxes using a terrestrial $^3He/^4He$ end-member value of $1x10^{-8}$.

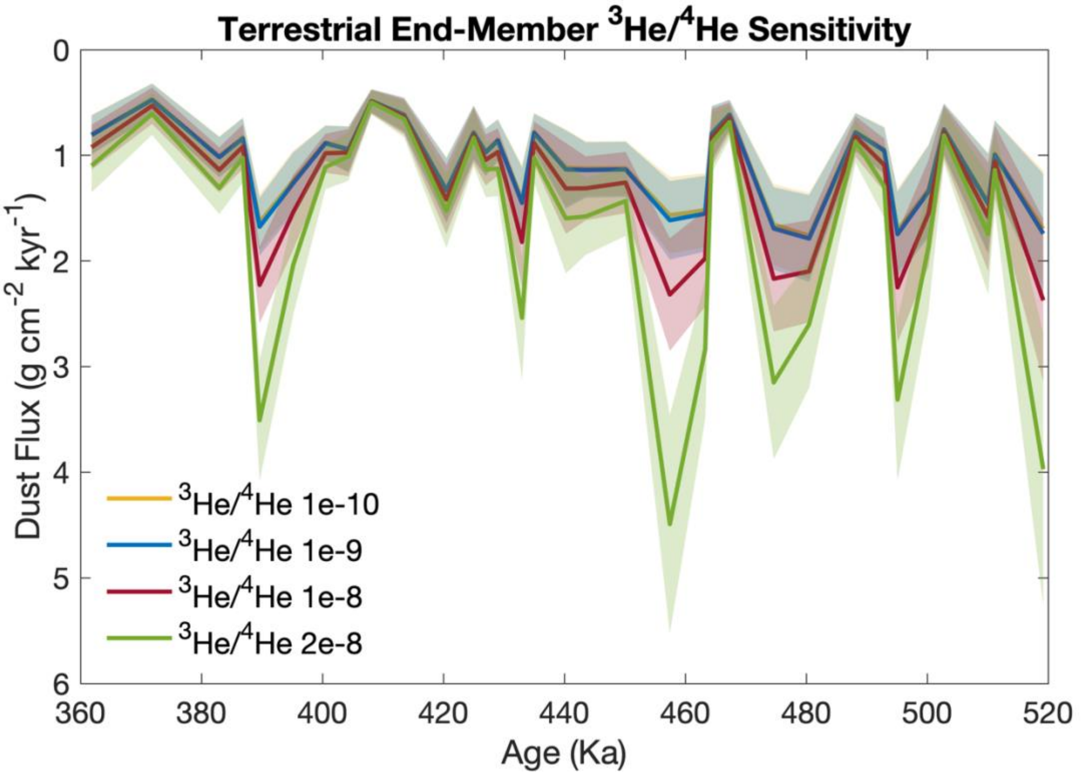

Figure S11. Sensitivity test of $^3He_{ET}$-normalized dust flux to the choice of terrestrial $^3He/^4He$ end member. While the magnitude of dust flux variability changes as a function of the end-member ratio, the overall pattern observed through time is robust and thus does not impact any of the conclusions of this study.
