# Supplementary material for: Pleistocene drivers of Northwest African hydroclimate and vegetation
Source: Nat Commun. 2022 Jun 21;13:3552. doi: 10.1038/s41467-022-31120-x (PMC9213457; doi:10.1038/s41467-022-31120-x)
Supplement: Supplementary file 3 — Description of Additional Supplementary Files [file 41467_2022_31120_MOESM3_ESM.pdf]

Description of Additional Supplementary Files

Filename: Supplementary Data 1
Description: Age model tie points for MD03-2705 based on $\delta^{18}O$ stratigraphy and magnetic reversal ages

Filename: Supplementary Data 2
Description: MD03-2705 alkane concentrations and isotopic compositions

Filename: Supplementary Data 3
Description: MD03-2705 helium isotopic compositions and dust fluxes

Filename: Supplementary Data 4
Description: MD03-2705 helium and thorium normalization comparison data
